# Supplementary material for: Study of Physico-Chemical Properties of Dough and Wood Oven-Baked Pizza Base: The Effect of Leavening Time
Source: Foods. 2023 Mar 26;12(7):1407. doi: 10.3390/foods12071407 (PMC10093465; doi:10.3390/foods12071407)
Supplement: Supplementary file 1 [file foods-12-01407-s001.zip › foods-2262376-supplementary.pdf]

Supplementary materials

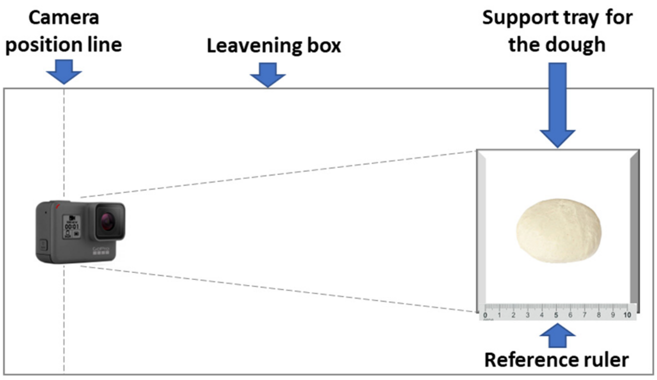

Figure S1 – Schematic representation of camera position with respect to the dough for image acquisition during leavening in the *ad hoc* plastic box.

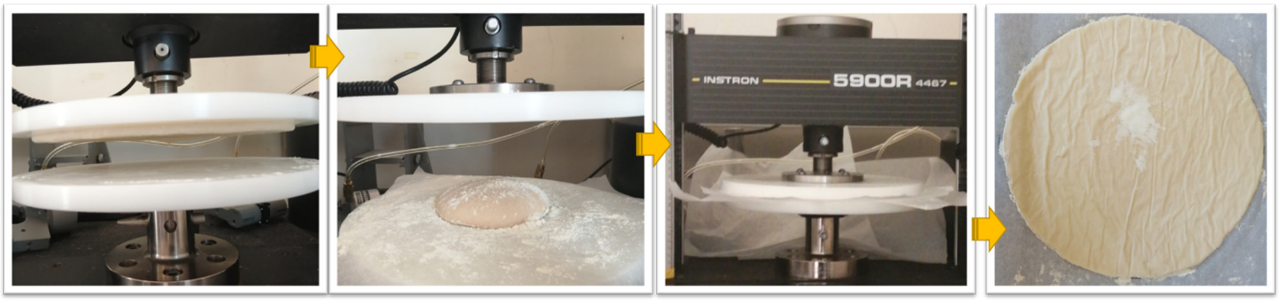

Figure S2 – Flow chart of pizza dough compression in the two mounted plates (Ø 30 cm) of the dynamometer (Instron, mod. 5900R, Norwood, MA, USA).

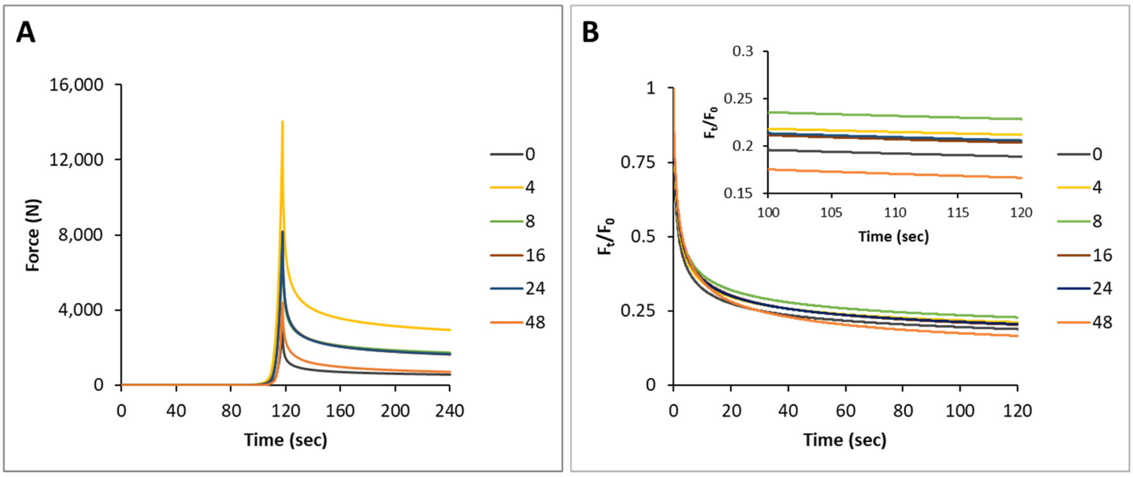

Figure S3 – Stress-relaxation (A) and elaborated relaxation graph (B) of pizza dough at selected leavening time.
